# Supplementary material for: Guiclupea superstes, gen. et sp. nov., the youngest ellimmichthyiform (clupeomorph) fish to date from the Oligocene of South China
Source: PeerJ. 2021 Jun 1;9:e11418. doi: 10.7717/peerj.11418 (PMC8176909; doi:10.7717/peerj.11418)
Supplement: Supplemental Information 1 — List of the morphological and meristic characters. Data matrix. Cladogram [file peerj-09-11418-s001.docx]

**SUPPLEMENTARY INFORMATION TO:**

**The youngest ellimmichthyiform (clupeomorph) fish to date from the Oilgocene of South China**

Gengjiao CHEN^1^, Mee-Mann CHANG^2,3^, Feixiang WU^2^, Xiaowen LIAO^1^

^1^Natural History Museum of Guangxi Zhuang Autonomous Region, Nanning 530012, China;

^2^Key Laboratory of Evolutionary Systematics of Vertebrates, Institute of Vertebrate Paleontology and Paleoanthropology, Chinese Academy of Sciences, Beijing 100044, China;

^3^College of Earth and Planetary Sciences, University of Chinese Academy of Sciences, Bejing 100049, China

**Appendix 1S**. List of the morphological and meristic characters used in phylogenetic analysis. Characters are mainly adopted from Chang and Maisey 2003 (CM), and Murray and Wilson 2013 (MW). Number following the abbreviation of the publications indicate the character number used in the publications.

1. Dorsal outline at dorsal fin origin: [0] smooth; [1] forming a marked angle or apex. (MW1).

2. Parietals: [0] meeting at midline; [1] separated completely by supraoccipital. (CM4, MW2).

3. Supratemporal commissure sensory canal passing through parietals: [0] absent; [1] present. (CM6)

4. Supraoccipital crest: [0] low and small; [1] high. (MW3).

5. Ornamentation on skull roof bones: [0] absent; [1] present. (MW4).

6. Pre-epioccipital fossa or fenestra: [0] absent; [1] present. (CM3).

7. Recessus lateralis: [0] absent; [1] present. (CM1, MW8).

8. Supramaxillary bones: [0] two; [1] one or none. (CM8, MW9).

9. Basipterygoid process of parasphenoid: [0] absent; [1] present. (CM11, MW10).

10. Tooth patch on posterior part of parasphenoid: [0] absent; [1] present. (CM10, MW11).

11. Beryciform foramen within anterior ceratohyal: [0] absent; [1] present. (CM12, MW14).

12. Foramen within posterior ceratohyal: [0] absent; [1] present. (MW15).

13. Number of branchiostegal rays: [0] more than sixteen; [1] less than sixteen.

14. Teeth on entopterygoid: [0] absent; [1] present. (CM9, MW16).

15. Dorsal process of posttemporal: [0] slender and sharp; [1] sub-rectangular; [2] broad, wider at distal tip than at midpoint of bone. (MW23).

16. Shape of cleithrum: [0] L-like (with a single angle in the bone); [1] S-like (with two angles); [2] almost straight (no angles). (MW22; Marrama and Carnevale, 2016, character 21).

17. Number of vertebrae excluding ural centra: [0] less than 50, [1] more than 50 (MW17).

18. Halves of neural arches of most abdominal vertebrae: [0] separated medially; [1] fused medially. (MW18).

19. Pleural ribs: [0] all ribs articulate with parapophyses; [1] anteriormost ribs articulate with deep pits on lateral side of all abdominal centra and those located posteriorly articulate with well-developed parapophyses; [2] all ribs articulate with deep pits on lateral side of all abdominal centra. (MW19).

20. Epineurals and epipleurals in caudal region: [0] absent; [1] present. (MW20).

21. Epicentrals: [0] absent; [1] present. (MW21).

22. Position of pelvic-fin insertion: [0] anterior to the dorsal fin origin; [1] about opposite to the dorsal fin base; [2] far behind dorsal fin base and closer to caudal fin base than to pectoral fin insertion.

23. Both dorsal and anal fins very posteriorly placed: [0] absent; [1] present.

24. Number of anal fin rays: [0] less than twenty [1] twenty or more. (MW24).

25. Number of hypurals: [0] six or more; [2] less than six. (CM21, MW26).

26. First hypural articulating with first ural centrum: [0] present; [1] absent. (CM27, MW28)

27. Proximal end of hypural 1: [0] massive; [1] sharp. (MW29).

28. Hypural 2: [0] autogenous; [1] fused to first ural centrum. (CM22, MW27).

29. Third hypural: [0] not expanded posteriorly leaving a gap or notch between second and third hypurals; [1] expanded posteriorly, leaving no gap or notch between second and third hypurals. (CM26, MW31).

30. Size of first ural centrum (diural terminology): [0] about equal with first preural centrum; [1] much smaller than first preural centrum. (CM18, MW33). **We coded Ornategulum as 1, according to Forey 1973, fig. 7.**

31. Number of uroneurals: [0] three or two; [1] one. (MW34).

32. First uroneural: [0] extending forward to second preural centrum; [1] failed to extend to second preural centrum. (CM25, MC32).

33. Fusion of first uroneural and first preural centrum: [0] absent; [1] present. (CM24, MW36).

34. First uroneural bearing a dorsal expansion of laminar bone: [0] absent; [1] present. (MW37).

35. Parhypural: [0] base/arch of bone fused with preural centrum 1; [1] autogenous. (CM20, MW39).

36. Number of epurals: [0] three; [1] less than three. (CM23, MW41).

37. Caudal scutes: [0] absent; [1] present. (CM17, MW43).

38. Predorsal scutes: [0] absent; [1] present. (CM14, MW46).

39. Predorsal scute series: [0] incomplete (absent in anterior part); [1] complete. (CM14, MW47)

40. Sub-rectangular scutes in anterior part of predorsal series: [0] absent; [1] present. (MW48).

41. Subrectangular scutes in the posterior part of predorsal series: [0] absent; [1] present. (MW49). **State 1 is applied to** ***Sorbinichthys africanus* and *S. elusive* because two of their most posterior scutes are elongate lateral extensive (Murray & Wilson 2011 p7).**

42. Series of spines on posterior margin of lateral wings of predorsal scutes: [0] absent; [1] present. (MW50).

43. Prominent median strong spine on posteriormost predorsal scutes: [0] absent; [1] present. (MW51)

44. Size of scutes of predorsal series: [0] all scutes of same size; [1] irregular in size, size of scutes increasing posteriorly. (MW52). **Following Murray and Wilson 2013 to consider state 1 ought to apply even if there is only a single irregularly shaped scute, we code *Sorbinichthys africanus* and *S. elusive* as 1**.

45. Predorsal scutes surface with radial ridges: [0] absent; [1] present. (CM15, MW53).

46. Number of predorsals scutes: [0] six to nineteen; [1] twenty or more. (MW54).

47. Abdominal scute series: [0] absent; [1] present, with only the pelvic scute; [2] present, with non-pelvic abdominal scute also. (CM16, MW55, MW56)

48. Postpelvic abdominal scutes bearing very prominent and strong ventral spine: [0] absent; [1] present. (MW57).

49. Size of lateral wings of abdominal scutes: [0] small; [1] deep, extended upward and covering the abdominal cavity laterally for at least one quarter of the distance from ventral body edge to vertebral column. (MW58).

50. Shape of lateral wing of abdominal series scutes: [0] spine-like, with large spaces between wings of scutes; [1] wide or spatula-like, with wings of adjacent scutes contacting to each other for most of their length. (MW59).

51. Number of abdominal scutes: [0] one; [1] twelve to fifteen; [2] nineteen or more. (MW61).

52. Number of postpelvic scute: [0] 2-3; [1] six or more.

53. Postdorsal scute series: [0] absent; [1] present. (MW60).

54. Number of predorsal bones (supraneurals): [0] 10 or more; [1] 9 or fewer. (MW62).

55. lateral line scales: [0] present; [1] absent. (CM29).

**Appendix 2S/3S**. Data matrix used in phylogenetic analysis. Each matrix includes 55 characters and 40 taxa with alternative of *Chanos/Elops* as outgroup.

Matrix

| Taxa | 0000000001  1234567890 | 1111111112  1234567890 | 2222222223  1234567890 | 3333333334  1234567890 | 4444444445  1234567890 | 55555  12345 |
| --- | --- | --- | --- | --- | --- | --- |
| *Chanos_chanos*  *Elops saurus* | 0100000100  0000000000 | 0011000001  0001001001 | 0100011000  0100000000 | 01101100‒‒  00010010‒‒ | ------0---  ------0--- | ‒‒000/  ‒‒000 |
| *Armigatus alticorpus* | 00100?0010 | 1?10010001 | 0101001110 | 0000001100 | 0000002010 | 2101? |
| *Armigatus brevissimus* | 0010110011 | 1?11010001 | ?001001110 | 0100001100 | 0000002?10 | 2101? |
| *Armigatus dalmaticus* | 0?100??0?0 | 1?11010001 | 0100001100 | 0000001100 | 0000002010 | 2101? |
| *Armigatus namourensis* | 0010110011 | 1011010001 | 0101001110 | 0100001100 | 0000102010 | 2101? |
| *Armigatus_oligodentatus* | 00100?0010 | 1010010011 | 0100001110 | 0100001100 | 0000002010 | 2101? |
| *Chirocentrus dorab* | 0110011000 | 0010101101 | 1001011101 | 01101100-- | ------1-00 | 0-0?1 |
| *Codoichthys carnavalii* | 00111?00?0 | ???0000?11 | ?100000100 | 010000?110 | 0000002010 | 2101? |
| *Denticeps clupeoides* | 0110001100 | 0110000121 | 1001100100 | 11010100-- | ------2000 | 21000 |
| *Diplomystus birdi* | 0011010011 | ??1?0101?1 | 0101000100 | 010?000111 | 1100012110 | 2101? |
| *Diplomystus dentatus* | 0011010011 | 1011?101?1 | 0001001110 | 0100001111 | 1100012110 | 21010 |
| *Diplomystus dubertreti* | 0011?100?? | ???01101?1 | ?1010??100 | ?00000?111 | 110001211? | ??01? |
| *Diplomystus_shengliensis* | 00110?0010 | ??110?00?1 | ?00100?110 | 0000001111 | 1100012?00 | ??01? |
| *Diplomystus solignaci* | 1?110?0010 | ???1?10110 | 0100000110 | 00???0111? | 1?010?2111 | 2101? |
| *Ellimma branneri* | 00101?0010 | 1?11010?10 | ?100000100 | 0000001110 | 1011102111 | 2101? |
| *Ellimma_longipectoralis* | 10101??010 | ???0010110 | ?101?00100 | 010?000110 | 1011102110 | 2101? |
| *Ellimmichthys goodi* | 10101?00?? | 1??1110110 | 1100000100 | 0001001111 | 1011102111 | 2101? |
| *Ellimmichthys longicostatus* | 10101?00?0 | 1??1110110 | 1000000100 | 0001001111 | 1011102111 | 2101? |
| *Ellimmichthys maceioensis* | 1?101?00?? | ???1?10??? | ?100?00110 | 000000?110 | 101110211? | 2?01? |
| *Eoellimmichthys_superstes* | 10100?001? | ??11010110 | ?001?????? | ????0?1110 | 0011102110 | 1101? |
| *Ezkutuberezi_carmenae* | 1010??00?0 | ??10?10111 | ?001?00110 | 000001011? | 10111?2110 | 2?00? |
| *Gasteroclupea_branisai* | 00100?0??? | 1?110200?1 | 0010101000 | 0100000111 | 0010012000 | 2?00? |
| *Guiclupea terminalus* | 00110?00?0 | 1011110111 | ?001000110 | 0100001110 | 0000112110 | 2100? |
| *Kwangoclupea dartevellei* | 0011110010 | ??????0??1 | ?101?00100 | 0000000110 | 00010?210? | 2101? |
| *Odaxothrissa_vittata* | 0110011100 | 0010000101 | 1101011101 | 01101000-- | ------2010 | 21001 |
| *Ornategulum sardinoides* | 0010110010 | 1?01?01001 | ?1000101?1 | 00010000-- | ------0--- | --000 |
| *Paraclupea chedungensis* | 10101?0010 | 10110100?0 | ?100001100 | 0000001111 | 1011102111 | 2101? |
| *Paraclupea seilacheri* | 1011110010 | 1111110011 | ?100000110 | 000?001111 | 1011102111 | 2101? |
| *Rhomichthys_intoccabilis* | 1010110010 | 1?11010110 | 1100?00110 | 0100100110 | 1011102111 | 2101? |
| *Scutatoclupea applegatei* | 10111???10 | ???1110111 | ?200?01100 | 0100001111 | 1011102111 | 2011? |
| *Scutatoclupea bacchiai* | 00101????0 | ?????101?1 | ?20000?110 | ????0?0110 | 0011?02111 | 2011? |
| *Scutatuspinosus itapagipensis* | 00101?00?0 | 1010110111 | 0000000110 | 0100001110 | 0011102101 | 2100? |
| *Sorbinichthys africanus* | 10100?0010 | ??11200111 | 0101?00000 | 01000?0110 | 1111012010 | 1000? |
| *Sorbinichthys elusive* | 1?100?00?0 | ???1200011 | ?001000000 | 0000000110 | 1111012010 | 1000? |
| *Thorectichthys macrocensis* | 10101?0010 | 1011010111 | 0101000110 | 0000001110 | 0001002110 | 2101? |
| *Thoractichthys rhadinus* | 10100?0010 | 1011010111 | 0101000110 | 0000001110 | 0001002110 | 2101? |
| *Triplomystus noorae* | 1010110010 | ??11110111 | 1100001111 | 0001001110 | 1011102111 | 2111? |
| *Triplomystus oligoscutatus* | 1010110010 | ??11110111 | 1101001111 | 0001001110 | 1011102111 | 1111? |
| *Tunisiaclupea speratus* | 10101??010 | 1?11010011 | 0100000100 | 0001001111 | 1011002111 | 2101? |
| *Tycheroichthys dunveganensis* | 10100?0010 | ??10110111 | 01010???00 | 0100010110 | 1011002111 | 2101? |


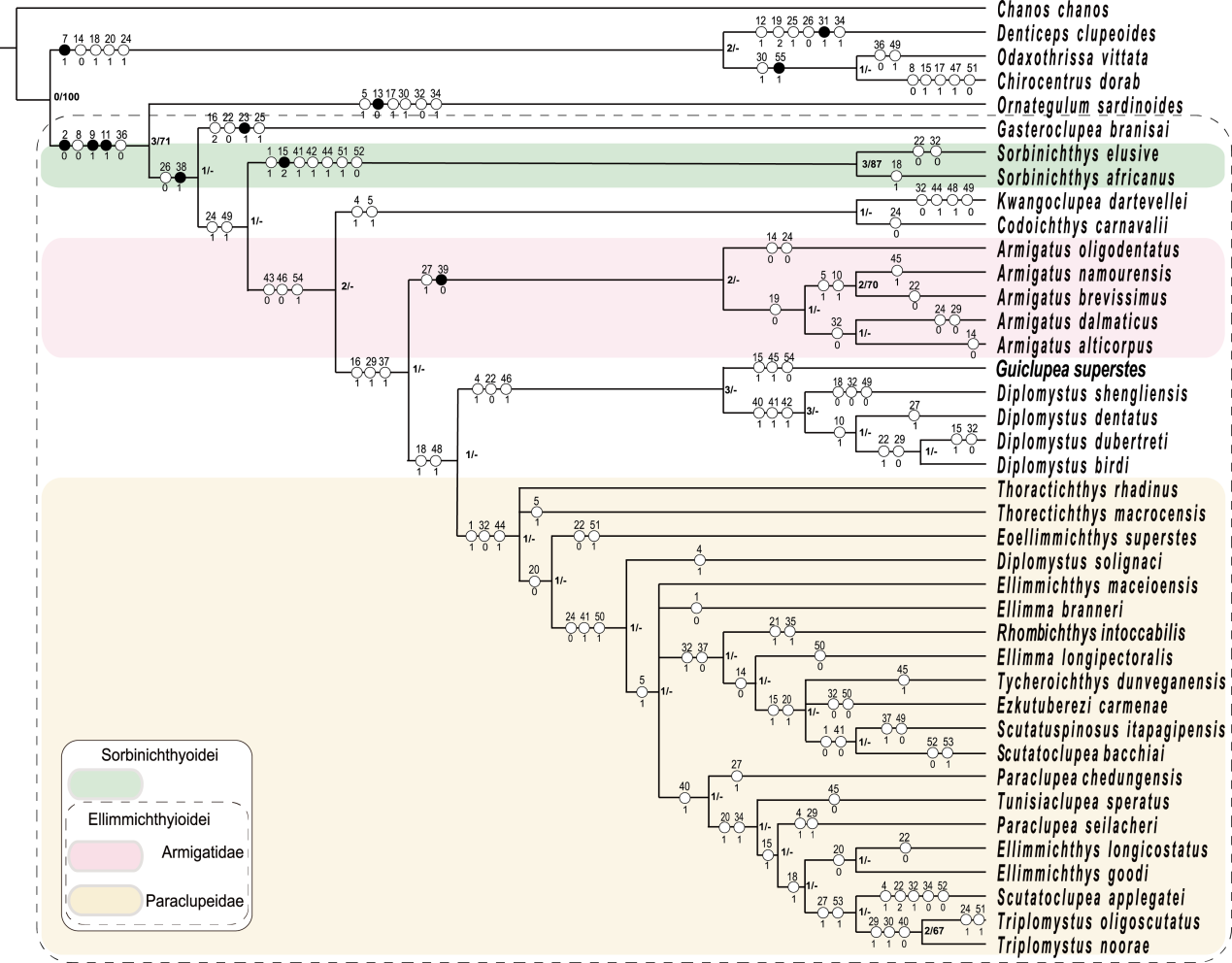


Figure 1S. The strict consensus tree of the four most parsimonious trees with *Chanos chanos* was used as outgroup taxon (tree length = 189 steps, CI = 0.323, RI = 0.670). Indexes in the right of nodes represent Bremer Index/Bootstrap support values that are higher than 50%. Numbers on each branch represent synapomorphies. Characters above black spot have consistency index of 1.


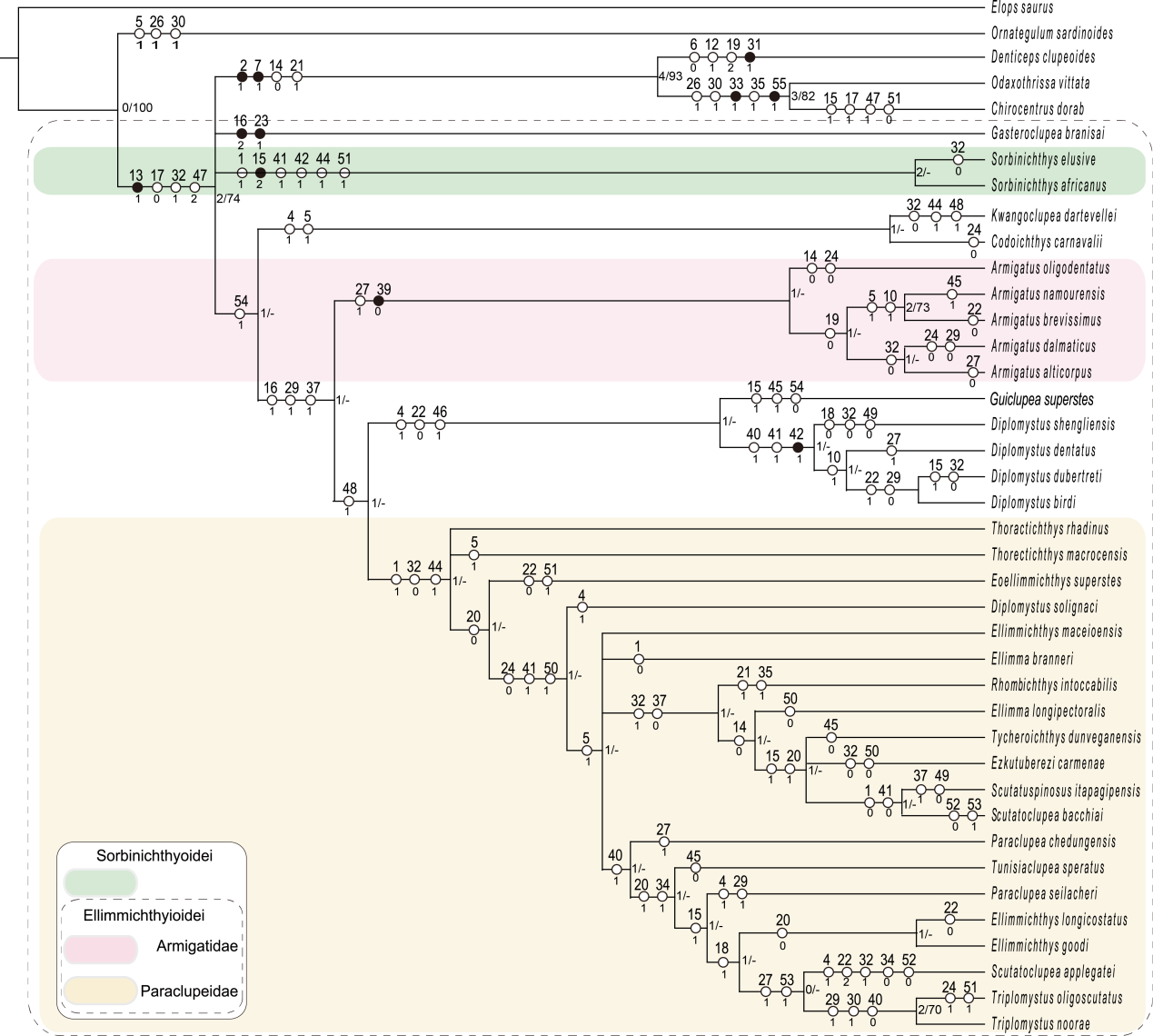


Figure 2S. The strict consensus tree of the sixteen most parsimonious trees with *Elops saurus* was used as outgroup taxon (tree length = 188 steps, CI = 0.324, RI = 0.671). Indexes in the right of nodes represent Bremer Index/Bootstrap support values that are higher than 50%. Numbers on each branch represent synapomorphies. Characters above black spot have consistency index of 1.


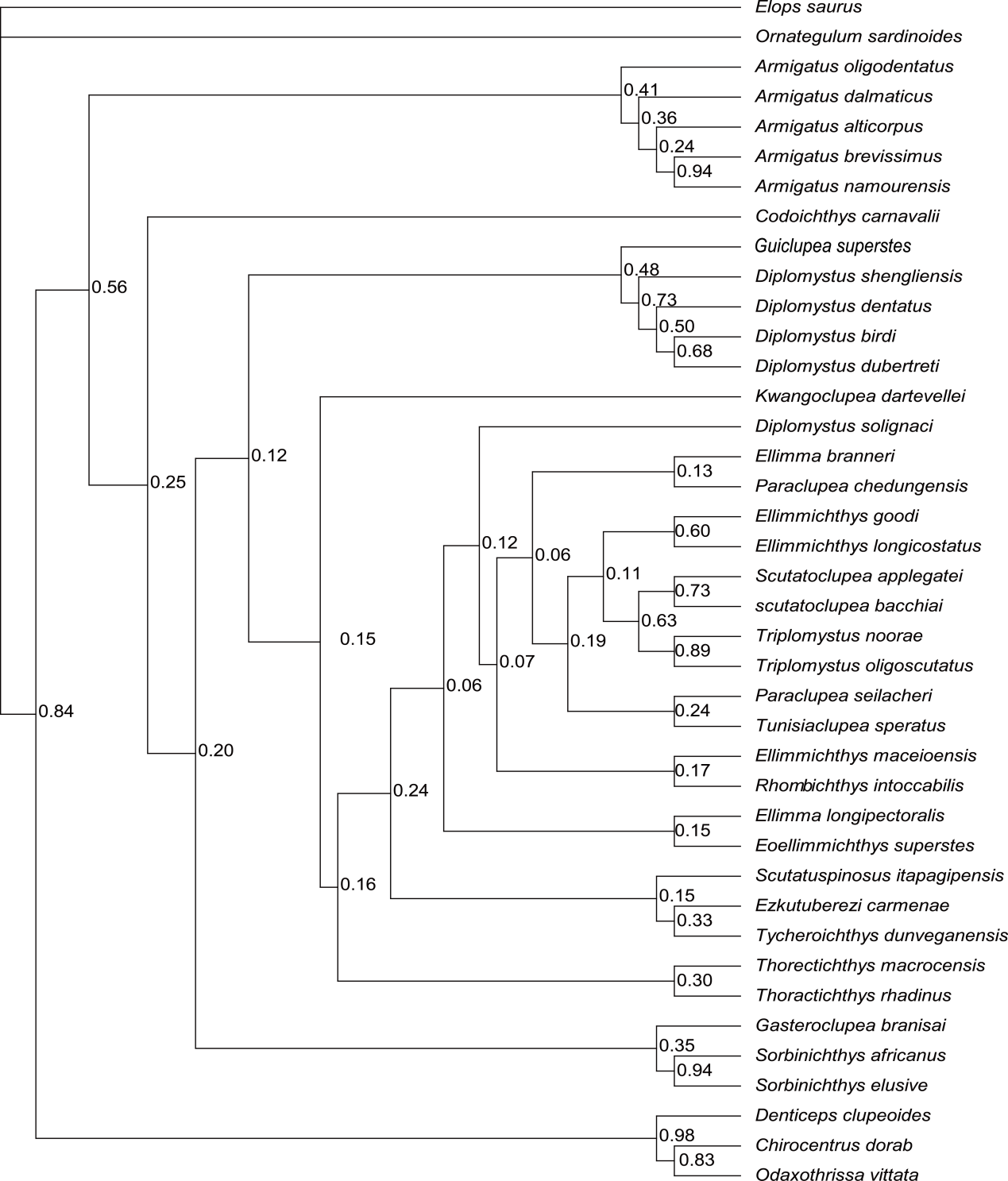


Figure 3S. Tree retrieved by Bayesian phylogenetic analyses with *Elops saurus* was used as outgroup taxon. The numbers at the internal nodes are the posterior probabilities of the corresponding clades.
